# Constructal thermodynamics combined with infrared experiments to evaluate temperature differences in cells

**DOI:** 10.1038/srep11587

**Published:** 2015-06-23

**Authors:** Umberto Lucia, Giuseppe Grazzini, Bartolomeo Montrucchio, Giulia Grisolia, Romano Borchiellini, Gianpiero Gervino, Carlotta Castagnoli, Antonio Ponzetto, Francesca Silvagno

**Affiliations:** 1 Dipartimento Energia, Politecnico di Torino, Corso Duca degli Abruzzi 24, 10129 Torino, Italy; 2 Dipartimento di Ingegneria Industriale, Università di Firenze, Via Santa Marta 3, 50139 Firenze, Italy; 3 Dipartimento di Automatica e Informatica, Corso Duca degli Abruzzi 24, 10129 Torino, Italy; 4 Dipartimento di Fisica, Università di Torino, via P. Giuria 1, 10125 Torino, Italy; 5 Dipartimento di Chirurgia Generale e Specialistiche, Banca della Cute, AOU Città della Salute e della Scienza Torino, Via Zuretti 29, 10126 Torino; 6 Dipartimento di Scienze Mediche, Università di Torino, corso A.M. Dogliotti 14, 10126 Torino; 7 Dipartimento di Oncologia, Via Santena 5 bis, 10126 Torino.

## Abstract

The aim of this work was to evaluate differences in energy flows between normal and immortalized cells when these distinct biological systems are exposed to environmental stimulation. These differences were considered using a constructal thermodynamic approach, and were subsequently verified experimentally. The application of constructal law to cell analysis led to the conclusion that temperature differences between cells with distinct behaviour can be amplified by interaction between cells and external fields. Experimental validation of the principle was carried out on two cellular models exposed to electromagnetic fields. By infrared thermography we were able to assess small changes in heat dissipation measured as a variation in cell internal energy. The experimental data thus obtained are in agreement with the theoretical calculation, because they show a different thermal dispersion pattern when normal and immortalized cells are exposed to electromagnetic fields. By using two methods that support and validate each other, we have demonstrated that the cell/environment interaction can be exploited to enhance cell behavior differences, in particular heat dissipation. We propose infrared thermography as a technique effective in discriminating distinct patterns of thermal dispersion and therefore able to distinguish a normal phenotype from a transformed one.

One of the fundamental abilities of the human body is its temperature control and regulation, which has been crucial for survival throughout human history^1^,^2^,^3^. A temperature variation of more than 3.5 °C from the mean resting body temperature of 37 °C could be fatal; indeed, for temperatures above 42 °C both the cellular cytoskeleton and organ and central nervous system function can be damaged^4^. Different strategies are involved in regulating body temperature and in maintaining physiological homeostasis; humans regulate their body temperature through a balance of heat production, absorption and loss^1^,^5^.

A temperature difference is required between cells and their environment, in order for them to survive^6^. For example, the related temperature gradient has been evaluated as approximately 0.4 °C cm^−1^ for *Streptococcus faecalis*^7^. This temperature difference is the driving force of the heat flows between cells and their environment across the membrane.

The internal temperature affects both the cell chemical reactions and heat and matter diffusion through the cell membrane^8^. Understanding how cells interact with and respond to the changes in their environment is essential for developing new approaches in biotechnology and medicine^9^,^10^. There is a continuous growing interest in applying Process Systems Engineering (PSE) techniques to biomedical problems^11^. For example, temperature is considered one of the fundamental factors in the analysis of cancer growth and a relationship between temperature and energy flow is evident when cellular metabolism is studied by analysing the substrate oxidative reactions^12^ conditioned by the temperature itself^12^,^13^. Therefore temperature measurement techniques represent a very interesting tool for cancer analysis. Recently, temperature analysis has been highlighted as an interesting topic of research in relation to the analysis of the behaviour of normal or cancer cells^14^. Although a variety of methods are available to measure body temperature, new techniques are required to compare the temperature variation related to the normal or deviant behaviour of cells systems.

Temperature variations on nanometre scales represents an essential topic of metrological innovation and technological development in many areas of biological, physical and chemical research and technology, with particular focus on temperature-induced control of gene expression and tumour metabolism in order to develop cell-selective treatments of diseases^15^,^16^,^17^,^18^,^19^.

Moreover, information obtained by nanoscale thermometry can be useful for engineering biological processes at the subcellular level^20^,^21^,^22^,^23^,^24^,^25^. Many approaches for temperature measurement at the nanoscale magnitude are currently being explored; for example, the scanning probe microscopy^26^, the Raman spectroscopy^27^ and fluorescence-based measurements using nanoparticles^28^ and organic dyes^29^. In particular, fluorescent polymers and green fluorescent proteins have recently been used for temperature mapping within a living cell^28^. However, these methods can be limited by some technical and experimental difficulties, as low sensitivity and systematic errors due to fluctuations in the fluorescence rate, the local chemical environment and the optical properties of the surrounding medium.

In order to compare the normal and altered behaviour of cells it is necessary to develop new techniques that allow information to be obtained with the scope of understanding the specific properties of distinct biological patterns.

Cell structure is related to its function and life is sustained by trapping and converting energy. Cells can perform this function by metabolic pathways, which are sequences of enzyme-catalysed reactions. Such pathways are strictly regulated, and this is essential to the existence of life because cells become disorganized and die without adequate control of metabolism. This regulation represents an efficient use of energy^14^.

In living cells, we can consider three kinds of energy flow essential to life processes:

-    Chemical, developed during the synthesis of complex biological molecules. Energy is needed to live, grow and create the internal molecular structure of the cells;
- Transport across the cell membranes, characterized by coupled inflow and outflow of ions, molecules and heat. Indeed, molecules and ions are transported across cell membranes against an electro-chemical or concentration gradient, by means of ion-pumping coupled with ATP-ase processes. Moreover, during the transport processes thermo-elastic and electric properties of the membrane are altered, with relative expenditure of energy;
- Mechanical, required to change the physical location of organisms, cells, and structures within cells.

The ultimate source of biological energy is the sunlight. Indeed, light energy is converted to chemical energy by phototrophs during photosynthesis. Plant and microbial producers, named autotrophic organisms, produce complex molecules that are subsequently used as a carbon source by chemoheterotrophs. These latter living systems use complex organic molecules as a source of molecules and energy to live and grow^30^. In order to survive, cells must efficiently transfer energy from their energy sources to their working systems. In living organisms the principal source of energy is adenosine-tri-phosphate (ATP). Energy can be converted into useful work by breaking down ATP to adenosine-di-phosphate (ADP) and orthophosphate (Pi). This is an energy-releasing reaction, which liberates free energy of about 50 kJ mol^−1^. The fundamental energy cycle of life in the cell consists of the conversion of chemical energy into work (for example when the energy stored in ATP is used for contraction of intracellular filaments or for active transport of molecules across membranes), followed by a new synthesis of chemical energy. The main mechanism of energy production is called oxidative phosphorylation (OXPHOS) and involves oxidation-reduction reactions in which electrons move down the potential gradient via the mitochondrial electron transport chain. During electron transfer, protons are pumped across the mitochondrial membrane to establish an electrochemical gradient. The energy stored in this gradient is used to drive ATP synthesis. For example we can consider the redox couple formed by the electron carrier nicotinamide-adenine-dinucleotide (NAD^+^) and oxygen^30^:


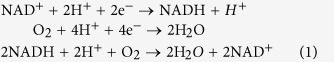


The free energy variation is related to the magnitude of the difference between the reduction potentials of the two reactions. When a two-electron transfer takes place there is a free energy change of 218.5 kJ mol^−1^ related to the electric negativity variation of the species. It is important to highlight that electrons transport plays a very important role in aerobic respiration, anaerobic respiration, and photosynthesis. Electron are transported due to the participation of carriers such as NAD^+^, flavin-adenine-dinucleotide (FAD) and flavin-mononucleotide (FMN), coenzyme Q (CoQ) and cytochromes, which use iron atoms to transport electrons by reversible oxidation and reduction reactions. The activity of the four multienzymatic complexes which constitute the respiratory chain coupled to the activity of the enzymatic complex known as ATP synthase are responsible for energy transformation. Enzyme activity is affected by the working condition related to environmental conditions^30^; consequently, the control of the environmental conditions is useful for checking cell metabolism and, as consequence, the normal or cancer cell behaviour^6^,^22^.

When normal cells evolve progressively toward a neoplastic state, they acquire a succession of new traits. Several divergences can be found between normal and transformed cells, such as alterations in metabolism, control of cell cycle, senescence and cell death, membrane composition, and transport across membranes. The entire range of modifications yields a new cellular phenotype, which behaves differently in its interaction and dependency on the microenvironment compared to normal differentiated cells^31^.

In order to investigate the relationship between cell behaviour and environment, and to identify metabolic processes responsible for differential responses to external stimulation in normal and cancer cells, it is of paramount importance to carry out studies with experimental settings that do not perturb the cell/environment interaction. The aim of this work is to distinguish normal cells from transformed cells evaluating thermal dispersion patterns, that should be different as suggested by constructal studies on exergy flows^14^. In order to achieve this goal, a new experimental set up is required to develop this type of analysis. In this article, infrared thermography is suggested as a new experimental approach to measure the temperature differences between normal and immortalized cells. We combine the constructal thermodynamic approach with the experimental analysis of heat dispersion, and we obtain evidences of a different thermal dispersion when distinct cell types are stimulated by the environment.

## Results

### The thermodynamic approach: cell flows and constructal law

As mentioned above, the transport across the membranes is crucial in cell metabolism. In order to develop a new open systems thermodynamic approach to cell metabolic behaviour it is fundamental to consider that transport phenomena can be effectively investigated by the application of the constructal law. Indeed, constructal law is a new approach introduced in thermodynamics to explain optimal shapes of natural configurations^32^,^33^,^34^,^35^,^36^,^37^,^38^.

In the cell, part of the energy is lost as heat outflow. Furthermore, when describing the modulation of metabolic pathways only the resultant biochemical molecules are known, without any information on the individual steps of biochemical and biophysical processes^6^. Therefore, a constructal approach has been introduced to analyse cell behaviour starting from the relationship between flows and irreversibility in cells^14^. Indeed, constructal theory highlights the fundamental role of flows across the border of a system in any thermodynamic process. Moreover, in this way we can also obtain information on the interactions between cells and environments, which consist of the actual flows across the cell membranes. To do so, we consider that cells are open and complex systems, and, as such, they can be analysed using the quantitative description of irreversibility by introducing the constructal approach to cell analysis and developing the quantitative calculation by using the concept of entropy generation^14^,^20^,^21^,^22^,^23^,^24^,^25^, defined as^33^:


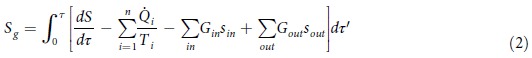


where *τ* is the lifetime of the process, which can be defined as the range of time in which the process occurs^20^,^21^,^22^,^23^,^24^,^25^; *Q* stands for the heat exchanged, *T* is the temperature of the thermal source, *s* represents the specific entropy and *G* is the mass flow. In relation to cells, the entropy generation has recently been evaluated as^20^,^21^,^22^,^23^,^24^,^25^:


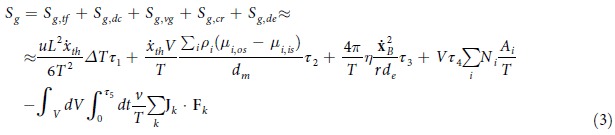


where

1. *S*_*g,tf*_ is the entropy generation due to the thermal flux driven by temperature difference;
2. *S*_*g,dc*_ is the entropy generation due to the diffusion current driven by chemical potential gradients;
3. *S*_*g,vg*_ is the entropy generation due to the velocity gradient coupled with viscous stress;
4. *S*_*g,cr*_ is the entropy generation due to the chemical reaction rate driven by affinity;
5. *S*_*g,de*_ is the entropy generation due to the irreversibility due to interaction with possible external fields present in the environment;

where *τ*_*i*_, *i* ∈ [1,5], is the lifetime of any process, being the time of observation the least common multiple of the processes lifetimes, and *L* is the typical length of a cell (which can be evaluated as its diameter if it is approximated as a sphere) and Δ*T* is the temperature difference between the cell and its environment; *μ*_*i*_ are chemical potentials of the *i*-th species, *V*_*m*_ and *d*_*m*_ are the volume and depth of the membrane, where the chemical potential gradient, 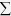
_i_*ρ*_*i*_(*μ*_*i*,*os*_ − *μ*_*i*,*is*_)/*d*_*m*_, occurs especially in cytoplasm, 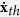 is the thermal velocity, *ρ*_*i*_ is the concentration of the *i*-th species and *os* and *is* stand for *outside* and *inside* the cell, respectively, while *T* represents the mean temperature of the membrane; *η* stands for the average viscosity coefficient, 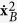 denotes the centre of mass velocity of all components in a cell, *d*_*e*_ the cytoplasm layer and *r* the mean cell radius; lastly, *N* is the number per unit time and volume of the *i*-th chemical reaction and *A* is the affinity, **F** is the force generated by the interaction with the external field and **J** stands for the associated flow. Moreover, the exergy of a system is defined as the maximum shaft work that can be carried out by the system and a specified reference environment, which is assumed to be infinite, in equilibrium, and ultimately to enclose all other systems: the environment is specified by its temperature, pressure and chemical composition.

Starting from these results a relationship between the temperature difference between the cell and its environment and the cell diameter was obtained as follows (Lucia, 2014a–f):


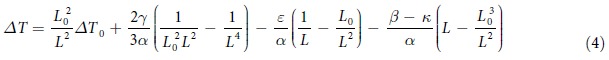


where *L*_0_ is the diameter of the daughter cell at its birth and Δ*T*_0_ is the temperature difference between the cell and its microenvironment at the outset; the constants in equation (4) are defined as follows^14^:

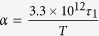
 , with *τ*_1_ in the range 15–269 ms.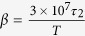, with *τ*_2_ ≈ 10 s.
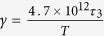
, with 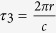 , with *c* ∼ 1540 m s^−1^.
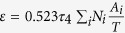
, with *τ*_4_ in the range of 17–1283 ns.
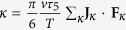
, with *τ*_5_ dependent on the interaction considered.

Furthermore, the volume of a cell is approximated by a mean cell sphere with the diameter,


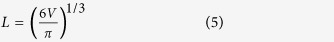


with *V* being the cell volume. The mean cell temperature can be assumed as *T* = 310 K; Δ*T* = 0.4 °C, but this quantity should be experimentally evaluated for different cell lines. The characteristic length can be evaluated as *L* = 2 *r*; the internal energy density can be evaluated as the ratio between the cell mean internal energy, which is considered the same as that of ATP, *U* = 3 × 10^−7^ J, and the mean volume of the cell is assumed to be *V* = 7600 *μ*m^3^, with the cell volume in the human body being in the range 200–15000 *μ*m^3^, which leads to *u* = 3.95 × 10^7^ Jm^−3^; the thermal molecular mean velocity inside the cytoplasm is considered to be 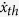 = 5 × 10^−5^ m s^−1^ and the membrane volume is evaluated as


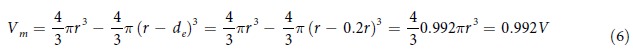


We note that the chemical potential gradient can be evaluated as the ratio between^14^ the mean value of the chemical potential *μ* = 1.20 × 10^−9^ J kg^−1^ and the membrane length *d*_*m*_ = 0.01 *μ*m, with the mean density ρ = 1000 kg m^−3^; the viscosity is evaluated as 6.91 × 10^−3^ N s m^−2^; *d*_*e*_ can be evaluated as *d*_*e*_ = 0.2 *r*; *η* ∼ 2.07 × 10^−3^ N s m^−2^ at 30 °C; 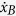 is evaluated as 3.0 × 10^−6^ m s^−1^.

The external fields are theoretically weighted by the constant κ. Their contributions depend on the type of fields considered, for example an electro-magnetic wave. Considering equation (4) we can argue that if external fields the coefficient (β − κ)/*α* becomes β/*α*, while if there are external fields it remains as (β − κ)/*α*. This variation in the coefficient determines a variation in the temperature difference Δ*T* − Δ*T*_0_. As a consequence of these thermodynamic analysis, it is reasonable to conclude that the temperature differences between cells that show distinct behaviour can be amplified by interaction between cells and external fields. This result is essential because it suggests two principles that may be relevant to the biomedical studies:

1. Operating on environmental characteristics amplifies the temperature differences between distinct cell types
2. Evaluation of different cellular phenotypes is possible by measuring the different amount of heat exchanged with the environment.

### Experimental evaluation of thermal dispersion by infrared thermography

We decided to apply the two aforementioned principles to an experimental set up in order to verify them. We changed the environmental characteristics by exposing the cells to ELF-EMF. The selected cellular models were primary fibroblasts and the immortalized NIH 3T3 cell line. The former is representative of a normal differentiated tissue; the latter is considered as a model of transformed proliferating precancerous tissue. Primary fibroblasts and NIH3T3 share the same mesenchymal origin. While primary fibroblasts have a limited life span, thus mimicking normal tissue self-renewal capacity, the NIH 3T3 are spontaneously immortalized cells established from disaggregated embryo fibroblasts in 1962 and have since become a standard fibroblast cell line. These two cell types have similar doubling time and are both contact inhibited. Primary fibroblasts and NIH3T3 are two cellular models used when comparing biological effects of pharmacological treatments^39^, in studies on skin re-epithelialization or wound healing^40^, when studying tissue engineered scaffolds^41^, or in nanotechnology biological applications^42^,^43^.

**Figure 1:**
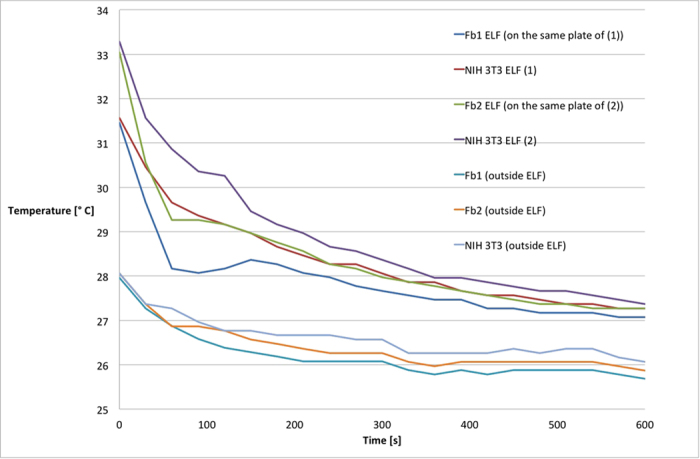
Thermal dispersion of cellular models. Thermal dispersion was evaluated at room temperature by the infrared thermographic camera. Two different preparations of primary fibroblasts (Fb1 and Fb2) and NIH 3T3 cells were analysed in the same conditions, either exposed for 6 days to electromagnetic field (ELF) or not exposed. The figure is representative of a set of three independent experiments.

**Figure 2:**
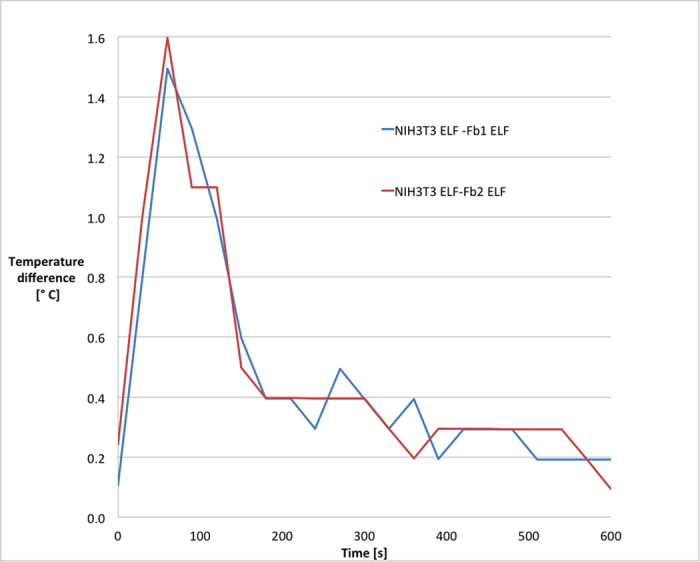
Differences in thermal dispersion of cells exposed to electromagnetic field. Two different preparations of primary fibroblasts (Fb1 and Fb2) and NIH 3T3 cells were analysed after 6 days of exposure to electromagnetic field. Values of Δ*T* were calculated for each well. Values of NIH3T3 cells were compared with values of primary fibroblasts seeded on the same plate. Temperature difference: *T*_NIH3T3_ − *T*_Fb_.

**Figure 3:**
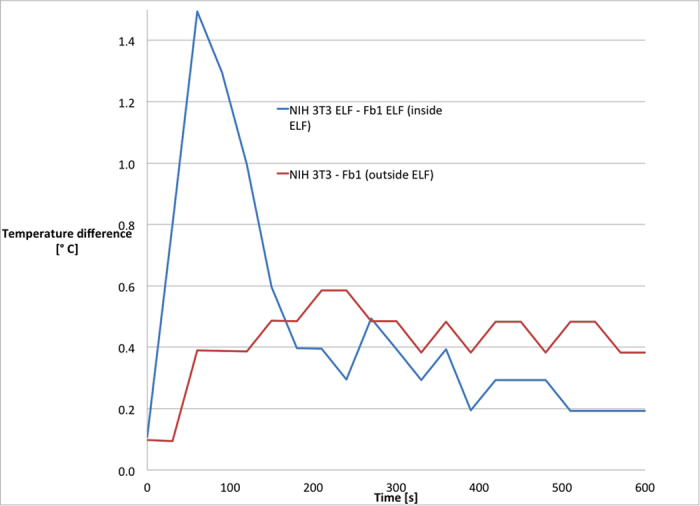
Differences in thermal dispersion of cells inside and outside the electromagnetic field. Primary fibroblasts (FB1) and NIH3T3 cells were analysed after 6 days of exposure to electromagnetic field or regular culture condition. Values of Δ*T* were calculated for each well. Values of NIH3T3 cells were compared with values of primary fibroblasts seeded on the same plate. Temperature difference: *T*_NIH3T3_ − *T*_Fb_.

**Figure 4:**
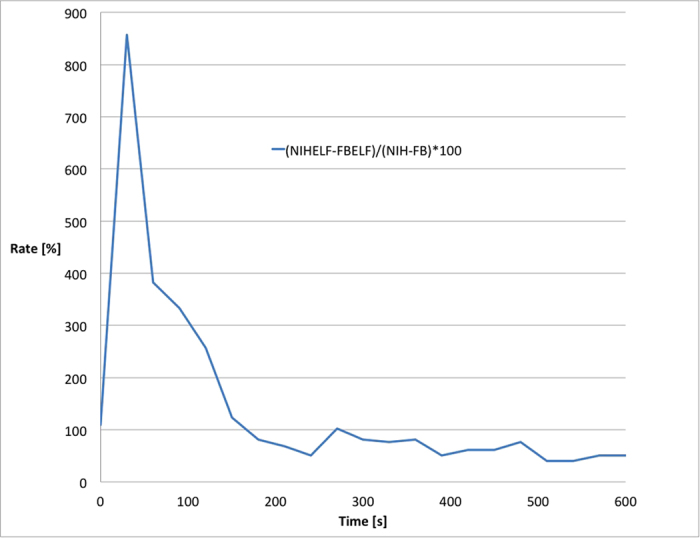
Thermal dispersion index of cellular models. Primary fibroblasts (Fb1) and NIH 3T3 cells were analysed after 6 days of exposure to electromagnetic field. Values of Δ*T* were calculated for each well. Values of NIH3T3 cells were compared with values of primary fibroblasts seeded on the same plate and expressed as percentage. The rate 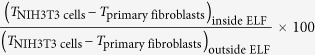 is represented.

By getting thermal infrared images we evaluated heat dispersion of cells, from 37 °C to room temperature. As shown in Fig. 1, differences in temperature drop were evident during the first 90 s. It is important to highlight that when cells were not exposed to electromagnetic field the pattern of heat dispersion was the same for the two cellular models. The difference in temperature between the two samples exposed to the electromagnetic field was calculated as Δ*T* = (*T*_NIH3T3 cells_ − *T*_primary fibroblasts_). Values of Δ*T* were calculated for each well. The values obtained for primary fibroblasts were compared with values relative to NIH3T3 seeded in the same plate. The results of this evaluation were plotted on graph, shown in Fig. 2, that demonstrated the identical pattern obtained from different experiments. From Fig. 2 it is also appreciable that the temperature difference between the two cellular models is highest in the first 90 s, and NIH3T3 cells in the first 120 s show a value of *T* higher than for primary fibroblasts on the same plate. From the data related to the cells exposed to the magnetic field, it is evident that NIH3T3 cells have a higher internal energy levels than the primary fibroblasts. The difference holds true only when cells are exposed to electromagnetic field; if not, the difference in temperature keeps constant at minimal values, as shown in Fig. 3. In order to quantify the magnitude of temperature differences triggered by magnetic field, we expressed such difference as:


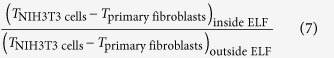


and obtained the graph shown in Fig. 4. This ratio is an indicator of how much primary fibroblasts are more efficient in thermal dispersion than NIH3T3 cells and it has been defined as the thermal dispersion index: this index represents the inability of the cells to fit their thermal power to environmental changes.

Primary fibroblasts display a high dispersion index, with a maximal value of 800% vs NIH3T3, which means that the primary fibroblasts adjust more efficiently their thermal production or dissipation than the NIH3T3 cells.

The results of this experimental approach demonstrate that selecting environmental conditions it is possible to appreciate distinct cellular phenotypes; these differences can be evaluated by thermal dispersion patterns measured by infrared thermography.

## Discussion

In this work we exploited a constructal approach to cell analysis and we developed an analytical approach by using the concept of entropy generation. Indeed, by referring to the constructal law, a living system presents two characteristics: it flows and it morphs freely toward configurations that allow all its currents to flow more easily over time. Life and evolution are a physics phenomenon, and they belong in physics. In a cell, a part of the energy is lost as heat outflow and only the resulting products of biochemical processes are known, while any individual step is inaccessible.

So, a constructal approach can represent a powerful theoretical method to analyse cell behaviour because the constructal theory highlights the fundamental role played by flows across the system’s border in any thermodynamic process. This can represent a new viewpoint in the analysis of the biochemical and biophysical behaviour of cells. Instead of studying the cell, a very complex system, we can now study how the cells exchange components and information with their environments, and the interactions between cells and environments, which consist of the flows across the cell membranes^44^,^45^. Therefore the spontaneous heat exchanged by the cell represents the interaction or, here, spontaneous communication between the cell and its environment. Lastly, it is obviously easier to access the environment than the living cell. Therefore, we decided to analyse the heat and mass flows across the membrane. The proposed thermodynamic model predicts that the temperature difference between cells with distinct metabolic characteristics can be amplified by an altered interaction with the external environment. The experiments carried out on cells exposed to ELF-EMF consolidate the thermodynamic approach. Through infrared thermography we were able to calculate a thermal dispersion index, which is high in the primary fibroblasts compared to immortalized NIH 3T3 fibroblast cell line (up to 800%). This significant difference implies that, when exposed to selected environmental conditions, transformed cells dissipate heat more slowly than their normal counterpart. Considering the differences in metabolism, intracellular organization and membrane composition between normal and transformed cells, several processes could be involved in the diminished thermal dispersion observed in immortalized cells; for example, the increased metabolic rate partly dispersed as heat, or the unchanged metabolic rate coupled to a decreased energy dispersion as heat, or the decreased thermal dispersion due to differences in membrane composition, etc.

The novelty of this work consists in the experimental validation of principles^14^,^20^,^21^,^22^,^23^,^24^,^25^ evinced from the thermodynamic analysis^14^,^20^,^21^,^22^,^23^,^24^,^25^ of cellular systems. The constructal approach has described the importance of selective cell/environment interactions, which depends on cellular phenotype and can modulate heat dispersion, and the theory has been tested in the experimental setting. In doing so, we have demonstrated that the cell/environment interaction can be exploited to enhance cell differences, in particular heat dissipation. Another element of novelty of this work is the exploitation of the infrared thermography as a technique effective in discriminating distinct patterns of thermal dispersion and therefore able to distinguish a normal phenotype from a transformed one.

## Methods

### Cellular models

The NIH 3T3 fibroblast cell line is a spontaneously immortalized cell line. Upon becoming immortal, these cells exhibit changes in genotype (they are hypertriploid) and phenotype (for example the cell line can acquire anchorage independence or can lose the characteristic of contact inhibition), possibly resulting in modified reactions to external influences. The disruption of homeostatic mechanisms that regulate normal cell growth and proliferation is a hallmark of cancer; several lines of evidence implicate cellular immortalization as a prerequisite for cell transformation^39^. By contrast, primary fibroblasts maintain a regulated and limited cell growth, therefore representing a model of normal differentiated cells.

NIH 3T3 embryonic fibroblast cells were purchased from American Type Culture Collection (ATCC), USA. Dermal primary fibroblasts were obtained from *Banca della Cute*, Turin, Italy, and were used in early passages. Cells were cultured in Dulbecco’s modified Eagle’s medium (DMEM) supplemented with 10% *foetal bovine serum* and 1% antibiotics [penicillin-streptomycin (Sigma-Aldrich)] at 37 °C in a humidified atmosphere containing 5% CO_2_. Cells were seeded in 6-well plates and exposed to extremely low-frequency electromagnetic fields (ELF-EMFs) for six days.

### ELF-EMF Exposure System

The experimental setup was composed of two independent couples of coaxial coils made of 200 loops (formed of copper wire, 0.3 mm in diameter) each loop being 2.5 cm in length (with a resulting loop density of 8000 loops per meter) and wound into a tightly packed plastic frame. The frame had a cylindrical shape with an outer radius of 8 cm, and the distance between the two coaxial coil couples was 8 cm. The cell culture dish was placed in the central part of the apparatus for magnetic irradiation. The experimental setup was placed in a magnetic shielded box where the inside residual magnetic field measured around 1–2 *μ*T. The box was made of stainless steel and the shield was produced by an 2 mm thick inner layer of mu-metal and an outer layer produced by a special aluminium-free alloy provided by the company G-Iron of Arezzo (Italy). The inner coils (7.5 cm radius) were connected to an AC current generator. The AC current signal was a square wave beginning from zero up to a chosen positive value, at 50 Hz and a duty cycle of 50%, the upper value was fixed in order to provide an AC magnetic field of up to 12 *μ*T rms in the irradiation volume. The outer coils were supplied with a DC current and they provided a constant magnetic field of 45 *μ*T. This value was chosen because in the Turin area, and particularly in the building where the measurements were carried out, the average value of the magnetic field is about (45 ± 5) *μ*T. Therefore, the DC magnetic field inside the box reproduced, in a controlled way, the effect of the average magnetic field of the environment. In the setup irradiation volume, the point-to-point variation for the total magnetic field was up to ± 5% of the nominal value. Inside the box, the temperature was kept constant, varying only ± 0.1 °C, and all the measurements were carried out in controlled conditions of pressure and humidity.

### Infrared thermography

Infrared thermographic devices provide images that represent surface temperatures by recoiling and measuring the magnitude of infrared radiation naturally emitted by a surface. Currently, IR devices are able to resolve surface temperature differences of 0.1 °C. As previously highlighted the temperature difference between cells is usually greater that the sensitivity of the infrared devices (estimated in the order of 0.4 °C).

In particular a Fluke Ti25 was used. Accuracy is ± 2 °C, while thermal sensitivity (NETD) is, as previously reported, ≤0.1 °C at 30 °C (100 mK).

Temperature measurements were carried out outside the incubator in a box built with polystyrene, given its properties as thermal insulator, and the box was connected to the camera by a circular opening. Between experiments a gap of 15 minutes allowed thermal stabilization of the setting. Prior measurements, a supplementary check was performed by a snapshot in absence of sample, to exclude thermal ghost images due to previous samples.

As each uncooled microbolometer in the focal plane array (160 × 120 in this case) can produce slightly different values from each other (e.g. for vignetting), prior to carrying out measurements ten flat fields were shot. Flat fields were averaged in order to produce a simplified master flat-field, which was applied to the IR images.

**Figure 5:**
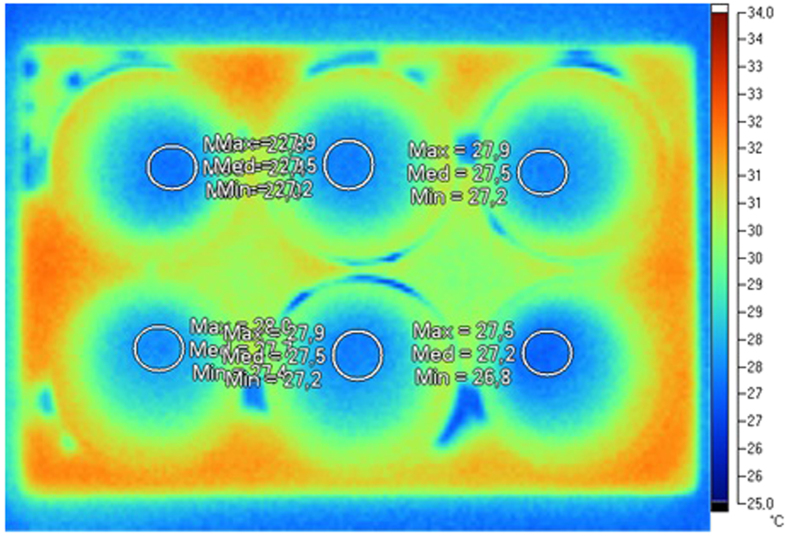
A sample of the infrared image.

As each well plate contained more than several hundred pixels and the central part of the well plate can be considered to have the same temperature, a number of about 70 pixels was selected (with a circular selection) inside each well plate, as depicted in Fig. 5. The selection was performed verifying that within the selection the standard deviation (among the selected pixels) was around 0.1 °C. This meant that it was possible to consider each selection (in a well plate) composed by a spatially ergodic signal, as a temporal average cannot be performed (temporal ergodicity) due to fast changing temperatures.

Given these experimental solutions, it was possible to detect temperature differences of 0.1 °C between two well plates in the same image. The reduced accuracy of the thermal camera and the possibly different initial temperature of the samples due to plate transfer from the incubator to the polystyrene box are therefore overcome.

### Thermal dispersion assay

Thermal dispersion was evaluated at room temperature by the infrared thermographic camera, which measured temperature variation from 37 °C to room temperature. Temperatures were sampled taking thermal images every 30 s for ten minutes, and data from each well were used to calculate Δ*T*. After the initial 90 s, cell temperature approached room temperature and the differences between samples declined. Data from different wells of the same plate were compared first as difference between Δ*T* of NIH3T3 cells and primary fibroblasts (temperature difference), and then as ratio of Δ*T* of NIH3T3 cells versus primary fibroblasts (dispersion index). Data from three independent experiments were analysed.

## Additional Information

**How to cite this article**: Lucia, U. *et al.* Constructal thermodynamics combined with infrared experiments to evaluate temperature differences in cells. *Sci. Rep.* **5**, 11587; doi: 10.1038/srep11587 (2015).
